# Kinesiophobia in patients with non-traumatic arm, neck and shoulder complaints: a prospective cohort study in general practice

**DOI:** 10.1186/1471-2474-8-117

**Published:** 2007-11-28

**Authors:** Anita Feleus, Tineke van Dalen, Sita MA Bierma-Zeinstra, Roos MD Bernsen, Jan AN Verhaar, Bart W Koes, Harald S Miedema

**Affiliations:** 1 Department of General Practice, Erasmus MC, Rotterdam, The Netherlands; 2 Netherlands Expert Center for Workrelated Musculoskeletal Disorders, Erasmus MC, Rotterdam, The Netherlands; 3 Department of Community Medicine, Faculty of Medicine and Health Sciences, United Arab Emirates University, Al Ain, United Arab Emirates; 4 Department of Orthopaedics, Erasmus MC, Rotterdam, The Netherlands

## Abstract

**Background:**

Complaints of arm, neck and shoulder are common in Western societies. Of those consulting a general practitioner (GP) with non-traumatic arm, neck or shoulder complaints, about 50% do not recover within 6 months.

Kinesiophobia (also known as fear of movement/(re)injury) may also play a role in these complaints, as it may lead to avoidance behaviour resulting in hypervigilance to bodily sensations, followed by disability, disuse and depression. However, in relation to arm, neck and shoulder complaints little is known about kinesiophobia and its associated variables.

Therefore this study aimed to: describe the degree of kinesiophobia in patients with non-traumatic complaints of arm, neck and shoulder in general practice; to determine whether mean scores of kinesiophobia change over time in non-recovered patients; and to evaluate variables associated with kinesiophobia at baseline.

**Methods:**

In this prospective cohort study set in general practice, consulters with a first or new episode of non-traumatic arm, neck or shoulder complaints (aged 18–64 years) entered the cohort. Baseline data were collected on kinesiophobia using the Tampa Scale for Kinesiophobia, the 13-item adjusted version: TSK-AV, and on patient-, complaint-, and psychosocial variables using self-administered questionnaires. The mean TSK-AV score was calculated. In non-recovered patients the follow-up TSK-AV scores at 6 and 12 months were analyzed with the general linear mixed model. Variables associated with kinesiophobia at baseline were evaluated using multivariate linear regression analyses.

**Results:**

The mean TSK-AV score at baseline was 24.8 [SD: 6.2]. Among non-recovered patients the mean TSK-AV score at baseline was 26.1 [SD: 6.6], which remained unchanged over 12- months follow-up period. The strongest associations with kinesiophobia were catastrophizing, disability, and comorbidity of musculoskeletal complaints. Additionally, having a shoulder complaint, low social support, high somatization and high distress contributed to the kinesiophobia score.

**Conclusion:**

The mean TSK-AV score in our population seems comparable to those in other populations in primary care.

In patients who did not recover during the 12- month follow-up, the degree of kinesiophobia remained unchanged during this time period.

The variables associated with kinesiophobia at baseline appear to be in line with the fear-avoidance model.

## Background

Complaints of arm, neck and shoulder are common in Western societies [1,2]. In the Netherlands, the 12 months prevalence in the general population has been estimated at 31% for neck pain, 30% for shoulder pain, 11% for elbow pain and 18% for wrist or hand pain [1]. The general practitioner (GP) is often consulted for these complaints [1,3,4]. In Norway 45% of adults experiencing non-inflammatory musculoskeletal pain reported consulting a GP within 12 months [3]. In persons with arm, neck and shoulder pain in the Netherlands this was about 30–40% [1].

A multi-disciplinary consensus was recently reached in the Netherlands to define upper extremity musculoskeletal disorders, to help professionals classify patients unambiguously and to improve communication amongst health care workers [5]. For the present study, we defined complaints as the symptoms for which a patient consults his/her GP, e.g. pain when active, pain in rest, tingling, stiffness, loss of strength, numbness, cold feeling in shoulder, arm or hand [5,6].

In the Netherlands, GPs are consulted 66 times annually per 1000 registered persons for a new complaint or new episode of neck or upper extremity complaints [4]. Despite treatment of these complaints, many patients do not completely recover within 3, 6 or 12 months after the first consultation. Previous work in the present population of non-traumatic arm, neck and shoulder complaints showed that, 46% of the patients still reported non-recovery after 6 months [7]. Similar results were found after 6 months in studies on shoulder pain [8,9]. Another study on neck and shoulder complaints reported 24% complete recovery after 3 months increasing to 32% after 12 months [10]. In a study on elbow complaints 13% reported complete recovery and 24% much improvement at 3 months compared with 34% and 21%, respectively, at 12 months [11].

Non-recovery in complaints of arm, neck and shoulder may be explained through the cognitive-behavioural oriented model for persistence of pain [12]; this model has been validated in chronic low back pain. Here, kinesiophobia, (also known as fear of movement/(re)injury) may lead to avoidance behaviour resulting in hypervigilance to bodily sensations, followed by disability, disuse and depression which may lead to a vicious circle of fear and avoidance in patients experiencing pain. This is in contrast to non-catastrophizing patients in whom not pain-related fear but rather a rapid confrontation with daily activities is likely to occur, leading to faster recovery. In support of this model, studies on patients with chronic low back pain reported that patients with higher levels of pain-related fear, have higher scores on pain and disability [12-15]. Furthermore, studies on acute low back pain and osteoarthritis in primary care have confirmed the relation between fear avoidance and disability [14,16,17].

In contrast to low back pain, for non-traumatic complaints of the arm, neck and shoulder little is known about the degree of kinesiophobia as measured with the Tampa Scale for Kinesiophobia [18] and its associated variables [12,17].

So far, no studies have investigated whether kinesiophobia remains stable during the transition period from new episode to chronic complaint. However, we expect the mean kinesiophobia scores to remain stable over time, because kinesiophobia was not specifically intervened upon. In addition, we expect that those variables of the fear-avoidance model involved in low back pain will also be associated with kinesiophobia in the case of non-traumatic arm, neck and shoulder complaints.

The aims of the present study were: (1) to examine the degree of kinesiophobia in patients with non-traumatic complaints of arm, neck and shoulder in general practice; (2) to establish whether the mean scores of kinesiophobia change over time in non-recovered patients; and (3) to evaluate variables associated with kinesiophobia in these patients at baseline.

## Methods

### Design and setting

The present study was part of a larger prospective descriptive cohort study which was performed in the Southwestern region of the Netherlands in 21 general practices.

During the 12- month study period, individual patient data were collected using self-administered questionnaires.

### Subjects

A total of 36 GPs from 21 practices recruited eligible patients from September 2001 through December 2002.

Inclusion criteria were: patients who visited their GP with a new complaint or new episode of complaints of neck, upper back, shoulder, upper arm, elbow, forearm, wrist or hand [6], age 18 through 64 years, and able to complete Dutch language written questionnaires. The episode was considered 'new' if patients had not visited their GP for the same complaint during the preceding 6 months.

Excluded were patients for whom the presented complaint could be explained by a trauma, fracture, malignancy, amputation, prosthesis, congenital defect or existing systemic and/or generalised neurological disorder and patients without a baseline score on the Tampa Scale for Kinesiophobia (TSK).

For the question on non-recovered patients, an additional inclusion criterion was applied, i.e. patients had to report on non-recovery. When they reported being "worse than ever" to "slightly improved" on a 7-point ordinal scale at both 6 and 12 months follow-up, they were considered to be non-recovered. Patients scoring "much improved" or "completely recovered" were considered to be recovered.

The Medical Ethics Committee of the Erasmus Medical Center in Rotterdam approved the study protocol. Written informed consent was obtained from all patients.

### Procedures

During the first consultation, patients received from their GP the study-information, an informed consent form, and the baseline questionnaire. A fax was sent by the GP to the investigators with a patient ID number, and information on age, gender, diagnosis and expected prognosis. After the research team received the completed informed consent form and the baseline questionnaire (within 8 weeks), inclusion criteria were verified in the computerized medical records. After inclusion, two follow-up questionnaires were sent from the research centre, one at 6 months and another at 12 months after the first consultation. All three questionnaires were self-administered.

#### (1) Tampa Scale for Kinesiophobia

Kinesiophobia was measured using the Dutch version of the TSK [18]. Each item is scored on a 4-point Likert scale with scores ranging from 1 "strongly disagree" to 4 "strongly agree." Good internal consistency, test-retest reliability and validity of the TSK in patients with low back pain and fibromyalgia have been demonstrated [12,19,20]. Originally, the TSK consisted of 17 items, including 4 reversed items. However, recent studies showed that the 4 reversed items had weak associations with the total TSK score and leaving out these items improved the factor structure of the TSK [18-20]. Therefore, we omitted these reversed items and used the adjusted version of the TSK with 13 items (TSK-AV). The total score can range from 13 to 52, with a higher score indicating a higher degree of kinesiophobia.

The TSK-AV was measured at baseline in all patients, and at 6 months and 12 months; at the two latter follow-up moments only patients who were not recovered had to complete the TSK-AV.

#### (2) Changes over time in non-recovered patients

**Figure 1:**
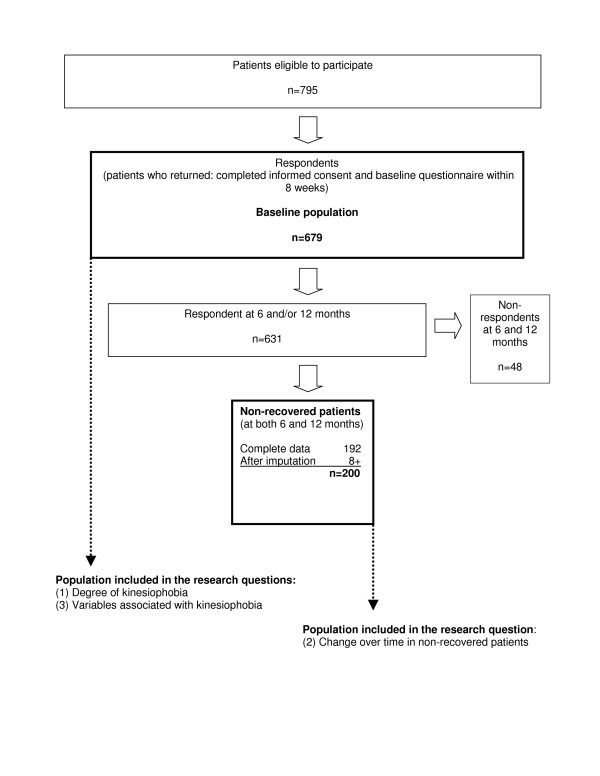
Flow-chart showing progression of the study.

Patients were included when they reported non-recovery at both 6 and 12 months follow-up (Fig. 1).

#### (3) Variables possibly associated with kinesiophobia

In the current study the variables explored were based on variables previously investigated in chronic (low back) pain [12,15]. Also included were other variables that seemed necessary based on the fear-avoidance model: i.e. active participation in sports (confrontation), musculoskeletal comorbidity (fear due in part to other complaints), recurrent complaint (good/bad previous experience with avoidance or confrontation), whether or not the complaint was located at a single site (thus more controllable), whether it matters where the complaints are located, and whether catastrophizing is the most important variable or whether other psychosocial variables are more or equally strongly associated with kinesiophobia.

The following variables were included in the model:

##### Patient characteristics

Age, gender, education level (low: no education, primary school or lower vocational school; medium: lower or higher general secondary school level or middle vocational school; high: higher vocational school or university), having paid work ('yes' when the question 'are you currently (self-)employed' was answered with the affirmative), and doing sports ('yes' when 'at least one hour a week actively performing sports intensely enough to became sweaty');

##### Complaint-specific determinants

Duration of the complaint before consulting the GP (0–6 weeks; 6 weeks-6 months; > 6 months), complaint severity in the previous week measured on an 11-point numerical scale from 0 (no complaints) to 10 (unbearable complaints). Disability of the arm, neck, shoulder or hand were measured with the Disability of Arm Shoulder and Hand (DASH)- questionnaire [21]. Each item was scored on a 5-point Likert scale. Response scores were summed and transferred to a score ranging from 0 (no disability) to 100 (completely disabled); a single- sited complaint (the patient indicated a single location on a manikin) versus multiple- sited complaint, location of the complaint (more than 1 is possible) and musculoskeletal comorbidity (other than the complaint consulted for), and recurrent complaints.

##### Psychosocial characteristics

Somatization and distress, both measured with the Four Dimensional Symptom Questionnaire (4DSQ), have been validated in Dutch populations in primary care [22]. A higher score indicates a higher degree of somatization or distress: Scores: low (0–10), medium (11–20), high (21–32).

Social support was measured with the Social Support Scale (SOS), a Dutch version of the 'Social Support Questionnaire' (SSQ) [23] and validated in Dutch primary care [24]. A higher score indicates a higher degree of social support.

Catastrophizing was measured with a subscale of the Dutch adaptation of the Coping Strategy Questionnaire (CSQ) [25,26], validated in a Dutch population. A higher score indicates a higher degree of catastrophizing.

An impression of health locus of control was assessed by one simple question 'Do you believe you can influence your health through your behaviour?' scored on a 4- point Likert-scale. The scores "considerable" or "to a large extent" were considered as 'yes' [27].

### Statistical analyses

#### (1) Degree of kinesiophobia

The mean TSK-AV score of the total population was calculated.

#### (2) Changes over time in non-recovered patients

In case a patient reported non-recovery at either 6 or 12 months and had a missing at the other follow-up time, 'belonging to the non-recovery group' was estimated using the multiple imputation technique [28]. This was done to decrease the possibility of selection bias. Multiple imputation assumes that data are missing at random (attrition depends on observed, not on unobserved variables) [29]. The computations were carried out with IVE ware (IVE ware, version 2.0, University of Michigan, USA, 2002). The variables univariately associated with non-recovery in this population (p < 0.10), extensively described in a previous study [7], were used for the imputation of non-recovery. We decided that when the value 1 (i.e. complaints) was imputed at least 6 times out of 10 for a particular patient, this patient would be considered as non-recovered.

The final group that reported non-recovery was used to analyze whether or not there was a change in the mean TSK score over 12 months. This was analyzed with the general linear mixed model, which takes correlation within subjects into account. No assumptions regarding the co-variance matrix were made. The analysis takes measurement error into account.

#### (3) Variables associated with kinesiophobia

Linear regression analysis was used to assess which determinants at baseline are related to kinesiophobia, with the TSK-AV score (range 13–52) as independent variable.

All independent variables were measured with a self-administered questionnaire. All scores were categorised. For possible determinants with clinically relevant classifications or predefined validated cut-offs, the existing cut-offs were used. If such cut-off points were not available for our population, the score was dichotomised according to the median score. Median scores were used for age, disability, social support and catastrophizing.

Variables that were univariately associated with kinesiophobia (p < 0.10) were selected for a multivariate analysis (step backward) procedure. When only one category of a categorical variable had a p-value less than 0.10, this variable also entered the model. The assumption of linear regression of homoscedasticity (constant variance of the residuals) was checked. Variables remained in the final model when they had a p-value < 0.05.

Differences between respondents and non-respondents regarding age, gender and recurrent or incident complaint, were analysed by multivariate logistic regression analyses (step backward). Variables remained in the final model when they had a significance level < 0.05.

Regression analyses were performed using SPSS version 11.0 (Chicago, IL, USA). Analyses of repeated measurements with the general linear mixed model were performed in SAS 8.2 (Cary, NC, USA).

## Results

A total of 795 patients fulfilled the criteria, of which 679 (85%) entered the cohort after they returned the completed questionnaire and informed consent form. The mean time between consultation and filling in the questionnaire was 2 weeks. Being a respondent was associated with older age (> 45 years) (odds ratio: 1.6; 95%CI: 1.0–2.3).

The number of patients entering the cohort and responding to the second (6 months) and third (12 months) questionnaire was 606 (89%) and 565 (83%), respectively. In total there were 48 non-responders on both the second and the third questionnaire.

### (1) Degree of kinesiophobia

At baseline, the mean score on the TSK-AV was 24.8 [SD: 6.2]. The median age of the study population was 45 years and 41% (n = 281) was men. The majority had paid work (78%) and less than half of the group practiced sports (44%). In 51% of the cases the GP was consulted within 6 weeks after onset of the complaint, 49% reported musculoskeletal comorbidity and 28% had endured the same complaint prior to the current episode.

Additional data on baseline characteristics are presented in Table 1.

**Table 1:** Baseline characteristics of the study population.

Variables		Internal missings *(n items)*	Total population (*n *= 679)

Patient characteristics			
Age (yrs) (18–64), *median (range)*		0	45.0 (18–64)
Male, *n (%)*		0	281 (41.4)
Educational level^a^, *n (%) *	low	0	243 (35.8)
	medium		242 (35.6)
	high		194 (28.6)
Having paid work,*n (%)*		0	532 (78.4)
Active sports participation, = 1 h/week, *n (%)*		0	302 (44.5)
Complaint- specific determinants			
Duration of the complaint, *n (%); n = 678 *	0-6 weeks	1	343 (50.6)
	6 weeks-6 months		161 (23.7)
	> 6 months		174 (25.7)
Severity in the last week (0–10), *median (range); n = 677*		2	6 [1–10]
Disability, DASH (0–100), *median (range); n = 678*		3	35.3 (2.6–99.1)
Comorbidity musculoskeletal, *n (%)*		0	330 (48.6)
Recurrent complaint, *n (%)*		0	191 (28.0)
Multiple- sited complaint		0	263 (38.7)
Location^†^, *n (%) *	neck	0	211 (31.1)
	upper back		53 (7.8)
	shoulder		374 (55.1)
	upper arm		86 (12.7)
	elbow		147 (21.6)
	forearm		41 (6.0)
	wrist		47 (6.9)
	hand		86 (12.7)
**Psychosocial characteristics**			
Kinesiophobia, Tampa-AV 13-item scale (13–52)	*median (range)*	0	24.0 (13–46)
	*mean (SD)*		24.8 (6.2)
Social support, SOS (12–60), *median (range)*		0	56.0 (26–60)
Catastrophizing, CSQ (0–60), *median (range); n = 678*		6	9.0 (0–53)
Somatization, 4DSQ (0–48), *n (%), n = 678 *	low (0–10)	21	500 (73.8)
	medium (11–20)		148 (21.8)
	high (21–32)		30 (4.4)
Distress, 4DSQ (0–48) *n (%)*	low (0–10)	0	430 (63.4)
	medium (11–20)		170 (25.0)
	high (21–32)		79 (11.6)
Yes, I can influence my health through my own behaviour, *n (%)*		0	401 (59.1)

SD: standard deviation; n = number of patients. ^a ^Educational level: low: no education, primary school or lower vocational school; medium: lower or higher general secondary school level or middle vocational school); high: higher vocational school or university); ^b ^More than one location is possible; DASH = disability of arm shoulder and hand questionnaire;4DSQ = four-dimensional symptom questionnaire; SOS = social support scale ;CSQ = coping strategy questionnaire; TAMPA-AV = Tampa adjusted version.

### (2) Changes over time in non-recovered patients

A total of 192 patients reported non-recovery at both 6 and 12 months. For 32 patients it was unknown at one point in time whether or not they were in fact non-recovered. After the multiple imputation procedure, 8 of these 32 patients were defined as non-recovered. This resulted in a total cohort of 200 non-recovered patients at 6 and 12 months as (Fig. 1).

In total, 48 patients were lost to follow-up at both 6 and 12 months. Mean score of the 48 dropouts on the TSK-AV (24.9 [SD: 5.6]) at baseline was similar to that of the 192 selected patients at baseline (26.0 [SD: 6.6]).

For the total of 200 non-recovered patients there was no significant change in kinesiophobia at 6 and 12 months compared to baseline (Table 2). When this analysis was repeated for the 192 non-recovered patients with complete data, the same result emerged.

**Table 2:** Change in kinesiophobia score in non-recovered patients during 12- months follow-up.

Time	Mean score	Estimate of changes in kinesiophobia *(n = 200) *β (95% CI)
Baseline	26.12	0
6 months	26.89	0.77 (-0.12, 1.65)
12 months	26.14	0.01 (-0.97, 1.00)

Results from general linear mixed model. Of the 200 non-recovered cases, 8 were defined as non-recovered after imputation. Of the total 600 observations (3 per case) 583 could be used in the analyses.

### (3) Variables associated with kinesiophobia

The results of the univariate and multivariate regression analyses are presented in Table 3. After multivariate regression analysis, 7 variables were significantly related to the score on kinesiophobia. Positive relations with kinesiophobia were found for a high degree of catastrophizing, a high degree of disability, and comorbidity of musculoskeletal complaints. Having a shoulder complaint was also related to a higher score on kinesiophobia. Further, low social support, high somatization and high distress contributed to the score, however the later two showed no clear dose- response relation with kinesiophobia.

**Table 3:** Associations with kinesiophobia: results of univariate and multivariate linear regression analyses.

	Total population (*n *= 679)		
		Univariate	Multivariate

Associated variables		B (95% CI)	B (95% CI)

Patient characteristics			
Older age (45–64 yrs)		0.67 (-0.26, 1.60)	
Male		0.33 (-0.61, 1.28)	
Educational level^a ^	low	0	
	medium	0.98* (0.01, 1.95)	
	high	-1.58* (-2.64, -0.56)	
Having paid work		-0.03 (-1.16, 1.10)	
Active sports participation, = 1 h/week		-1.14* (-2.07, -0.20)	
Complaint- specific determinants			
Duration of the complaint	0–6 weeks	0	
	6 weeks-6 months	-0.66 (-1.76, 0.43)	
	> 6 months	1.28* (0.21, 2.34)	
Complaint severity in the last week, score > 6		1.80* (0.86, 2.74)	
Disability, DASH score > 35.34		3.90* (3.01, 4.78)	2.78 (1.92,3.65)
Musculoskeletal comorbidity		2.38* (1.46, 3.29)	1.89 (1.05,2.74)
Recurrent complaint		1.08* (0.04, 2.11)	
Multiple- sited complaint		1.53* (0.58, 2.48)	
Location^b ^	neck	-0.00 (-1.01, 1.01)	
	upper back	0.82 (-0.92, 2.56)	
	shoulder	1.44* (0.51, 2.37)	0.86 (0.04,1.69)
	upper arm	-0.49 (-1.89, 0.91)	
	elbow	1.06 (-0.70, 2.19)	
	forearm	0.56 (-1.39, 2.52)	
	wrist	-0.73 (-2.57, 1.10)	
	hand	-0.15 (-1.55, 1.25)	
Psychosocial characteristics			
Low social support, SOS (12–60), score < 56		2.33* (1.37, 3.29)	1.17 (0.28,2.05)
Much catastrophizing, CSQ (0–60), score > 9		4.26* (3.39. 5.14)	3.15 (2.27,4.03)
Somatization, 4DSQ (0–32)	low (0-10)	0	0
	medium (11–20)	0.45 (-0.68, 1.57)	-1.27 (-2.35,-0.19)
	high (21–32)	6.84* (4.63, 9.05)	2.39 (0.19, 4.60)
Distress, 4DSQ (0–32)	low (0–10)	0	0
	medium (11–20)	0.78 (-0.30, 1.85)	0.07 (-0.96, 1.09)
	high (21–32)	4.22* (2.80, 5.64)	1.61 (0.12, 3.10)
Yes, I can influence my health through my own behaviour		-0.55 (-1.50, 0.40)	

n = number of patients; B = unstandardized coefficients; * p < 0.10; ^a^Educational level: low: no education, primary school or lower vocational school; medium: lower or higher general secondary school level or middle vocational school); high: higher vocational school or university); ^b^More than one location is possible; DASH = disability of arm shoulder and hand questionnaire;4DSQ = four-dimensional symptom questionnaire; SOS = social support scale ;CSQ = coping strategy questionnaire. Multivariate linear regression analysis: Adjusted R^2 ^= 0.24 (explained variance) and the intercept = 21.17; total number of complete cases, n = 675.

## Discussion

Recent studies in primary health care, reported mean TSK-AV scores similar to our study group; these populations consisted of patients with chronic neck pain [30], osteoarthritis [17], and acute low back pain [16]. However, in secondary care two studies on chronic low back pain reported mean TSK-AV scores of 31.6 [SD: 7.2] [19] and 33.8 [SD: 7.6] [18]. In the non-recovered patients in our study group the mean TSK-AV score at 12 months follow-up was 26.1 [SD: 7.8].

A possible explanation for differences in mean kinesiophobia scores between primary care populations and patients with chronic complaints at other care levels, might be that fear is a predictive factor in developing chronic complaints. This would imply, that patients who develop chronic complaints more frequently have a higher baseline score compared to quick recoverers. In previous work in the present population of patients with non-traumatic arm, neck or shoulder complaints, we found a univariate relation (odds ratio 1.4; 1.0 to 2.0) of the TSK-AV score (higher than the median score) with non-recovery at 6 months [7]. However, kinesiophobia did not contribute to the multivariate model on non-recovery [7].

Another study in general practice reported a small and only borderline significant effect of high fear avoidance predicting less future pain (at 3 and 12 months) and less functional disability (at 3 months) [10]. In both studies, other psychosocial variables (such as worrying and somatization) were more important predictors of poor outcome than kinesiophobia [7,10].

A study in physiotherapy practice in these complaints, reported that high kinesiophobia, high catastrophizing and high somatization were predictors of non-recovery [31]. Differences in the distribution of population characteristics may affect the importance of kinesiophobia as a predictor of outcome. At baseline, our population consisted of 58% women, compared to 71% in the study of Karels et al. [31], duration of complaints less than 6 weeks: 50% vs 24%; 6 weeks-6 months:24% vs 41%; and more than 6 months:26% vs 35%; specific diagnosis (59% vs 36%).

Besides, distribution of population characteristics, the time period can play a role as well. Bot et al. [10] reported that the psychosocial variables predictive of outcome at 12 months, are different from those predictive of outcome at 3 months; which was in line with the findings of both Boersma and Linton [32] and van der Windt et al. [33] who reported that associations of several psychological variables and outcome can be different in subgroups with a longer duration of complaints Therefore, no consistent conclusions can yet be drawn about the prognostic value of kinesiophobia and fear avoidance, in the outcome in non-traumatic arm, neck and shoulder complaints and its consequences for treatment.

Furthermore, van der Windt et al. reported that possible differences on scores may be due to the location of complaints as well. In their prognostic study in primary care low back pain patients scored higher on catastrophizing, distress and somatization, compared to patients with shoulder pain. However, scores on fear avoidance did not significantly differ [33].

In our study population there was no change over time on mean TSK-AV scores in patients who did not recover from complaints of arm, neck or shoulder. However, no definite conclusion about the stability of scores in the transition from a new episode to chronic complaint, can be based solely on this result. Although all patients had a new episode of complaints for which they had not consulted their GP in the previous 6 months, at baseline 25.7% of them already reported that they had endured their symptoms for more than 6 months.

In an intervention study on treatment of kinesiophobia in 6 patients with chronic low back pain [34], the scores in the 4-week baseline period also seemed stable. Here, kinesiophobia scores were only reduced by an exposure in vivo intervention (not during graded activity). In this chronic low back pain population influencing kinesiophobia seems to require specific treatment. Although the results of the latter study seem to be in line with our results, it should be noted that these low back pain patients were recruited in rehabilitation, with a median pain duration of 4 years, and had to have a relatively high score (> = 40) on the TSK to be included in the study [34].

Additionally, at baseline we found no multivariate relation of duration of complaint with the TSK-AV score. Time did not explain differences in the degree of kinesiophobia in primary care.

In our total population, at baseline, positive associations were found between kinesiophobia and a high degree of catastrophizing, a high degree of disability, and comorbidity of musculoskeletal complaints. Based on the 'fear- avoidance model' [12], the association between catastrophizing and the TSK score was expected, as also confirmed in patients with low back pain [13,19,20] and patients with chronic musculoskeletal and neuropathic diagnoses [20].

The association of disability, also part of the fear-avoidance model, has also been confirmed in other studies [15,16,18,20], some of which report on functional disability. Another study in patients with osteoarthritis [17], reported on an association between kinesiophobia and functional limitations. Although, disability and functional limitations are not exactly the same, they are connected. In the present study, we reported on disability measured with the DASH, which, according to its developers focuses on physical function. According to the International Classification of Functioning (ICF), the DASH mainly focuses on disability at the level of activity limitations, which is the domain of rehabilitation therapy [35].

A noteworthy finding was that the presence of musculoskeletal comorbidity was associated with a higher score on kinesiophobia. On closer inspection of the subgroup reporting musculoskeletal comorbidity (n = 330), we found that the majority also had low back pain, followed by a smaller group reporting osteoarthritis of hip or knee, and a few (n = 23) reporting comorbidity of arm, neck or shoulder. However, we have no information on the duration of this co-occurring musculoskeletal complaint. This raises the question, whether the higher score on kinesiophobia was mainly the effect of the concurrent chronic low back pain, or a previous negative experience in general. Although heterogeneity is the reality of the general practice population, we checked whether having co-morbidity modified the association between the variables in the final model and kinesiophobia. This was not the case.

Furthermore, most psychosocial variables remained in the final multivariate model. Pearson correlation coefficients between the variables included in the final model ranged from -0.21 to 0.47, of which the highest was for distress and catastrophizing. Thus, distress, somatization and social support, do not measure the same thing, and each variable has its independent association with kinesiophobia. However, catastrophizing showed the strongest association with kinesiophobia.

Because in the present study the area of possible complaints was extensive (compared with studies on e.g. low back pain) we also included location of complaints as a variable. The results show that complaints involving the shoulder were positively related to kinesiophobia; we have no clear explanation for this finding. A possible explanation may be that the shoulder is a large and central joint (compared to elbow, wrist and hand) providing stability and mobility in many stances and movements of the whole upper extremity. However, this was not confirmed by additional analyses in which we compared mean disability scores. Further, we did not find more musculoskeletal comorbidity among patients with shoulder complaints. Besides a true association, this association may partly be explained by a larger group size and accompanying smaller confidence intervals and smaller p-values compared with, e.g., complaints located at the elbow.

The present study has some limitations that need to be addressed. First, the questions on the TSK-AV relate to 'pain', whereas our patients reported on 'complaints' (as defined in the introduction) and not exclusively on pain. However, 675 (99%) patients reported pain when active and/or in rest, and only 4 patients, without pain, reported on tingling. Therefore, our results will also hold when excluding these 4 latter patients. Besides, although the cognitive-behavioural oriented model was developed for persistence of pain, the concept of avoidance behaviour may also be applicable in patients reporting other complaints, such as tingling. Nevertheless, no definite conclusions can be drawn on this matter. In patients with chronic fatigue syndrome however, fear- avoidance has also been reported [36]. After our inclusion period had started in September 2001, we found reports on an adjusted Tampa scale for patients with chronic fatigue syndrome where the term 'pain' had been replaced by 'symptoms' [36]. In the present study, replacing 'pain' by another term might have been a better option.

Since its development in chronic and later acute low back pain patients, the TSK has also been introduced in other populations (e.g. chronic fatigue syndrome [36], osteoarthritis [17], chronic neck pain [30], pain- free people [37]). In patients with non-traumatic arm, neck and shoulder complaints, the TSK has been used as a possible predictor in prognostic studies [7,11,31], and as outcome measure in randomised clinical trials in chronic neck pain [30]. So far, no studies have reported on the psychometric properties of the TSK in arm, neck and shoulder complaints. Although our mean score seems comparable to those in other primary care populations, and associated variables seem in line with other studies, future studies on psychometric properties need to confirm whether the TSK is a valid measurement instrument in this particular population.

Another limitation is that we used one simple question to give an indication of 'health locus of control', instead of using a validated multi-item questionnaire; therefore, the strength of the association should be interpreted with caution. However, the negative direction of the association was as expected, i.e. a higher degree of kinesiophobia was associated with less health locus of control.

Despite also being part of the fear avoidance model, we did not measure depression in our patients. Although we did include questions on several other psychosocial variables, we considered that the questions of the 4DSQ depression scale (e.g.: "During the past week, did you feel that life was meaningless?" "Did you feel that life is not worth while?" "Did you feel that you would be better off if you were dead?") were less appropriate in our population with new non-traumatic arm, neck and shoulder complaints. In the validation study of the 4DSQ, Terluin et al. reported that the applicability of their depression scale was limited in an unselected sample of primary care patients (n = 2,127) because of the low mean scores on depression, due to the relatively low prevalence of depressive disorders; they further concluded that the distress score that was measured, gives an indication of psychosocial dysfunctioning in general, including mild depressive symptoms [22]. However, including depression in our study would have yielded some additional information.

## Conclusion

The mean TSK-AV score in our population of patients with non-traumatic arm, neck and shoulder complaints seems comparable to those in other populations in primary care.

In patients who did not recover during the 12- month follow-up, the degree of kinesiophobia remained unchanged during this time period.

The variables associated with kinesiophobia at baseline appear to be in line with the fear-avoidance model.

Future studies are needed to provide more data on the psychometric properties of the TSK-AV and the prognostic value of kinesiophobia on outcome in this particular population.

## List of abbreviations used

GP: General practitioner;

TSK-AV: Tampa Scale for Kinesiophobia, 13-item adjusted version (TSK-AV);

SD: Standard deviation.

## Competing interests

The author(s) declare that they have no competing interests.

## Authors' contributions

AF collected the data, performed the analyses with TvD and wrote the article. AF had full access to all the study data and had the final responsibility for the decision to submit for publication.

TvD entered part of the data, performed the analyses with AF, and wrote the first draft of the article.

SMAB-Z was the principal investigator of the study and contributed to the design and coordination of the study and interpretation of the results.

RMDB performed part of the analyses.

JANV is an expert in the field of musculoskeletal complaints and contributed to the interpretation of the results.

BWK is an expert in the field of epidemiology and contributed to the design and coordination of the study and the interpretation of the results.

HSM is an expert in the field of musculoskeletal complaints and epidemiology and contributed to the design of the study and the interpretation of the results.

All authors declare that they have participated in the writing and editing of the manuscript and that they have read and approved the final version.

## Pre-publication history

The pre-publication history for this paper can be accessed here:


